# The role of computer-assisted radiographer reporting in lung cancer screening programmes

**DOI:** 10.1007/s00330-022-08824-1

**Published:** 2022-05-14

**Authors:** Helen Hall, Mamta Ruparel, Samantha L. Quaife, Jennifer L. Dickson, Carolyn Horst, Sophie Tisi, James Batty, Nicholas Woznitza, Asia Ahmed, Stephen Burke, Penny Shaw, May Jan Soo, Magali Taylor, Neal Navani, Angshu Bhowmik, David R. Baldwin, Stephen W. Duffy, Anand Devaraj, Arjun Nair, Sam M. Janes

**Affiliations:** 1 Lungs for Living Research Centre, UCL Respiratory, Rayne Institute, University College London, 5 University Street, London, WC1E 6JF UK; 2 Wolfson Institute of Population Health, Barts and The London School of Medicine and Dentistry, Queen Mary University of London, London, UK; 3 Department of Radiology, University College London Hospital, London, UK; 4 Department of Radiology, Homerton University Hospital, London, UK; 5 Department of Thoracic Medicine, University College London Hospital, London, UK; 6 Department of Thoracic Medicine, Homerton University Hospital, London, UK; 7 Respiratory Medicine Unit, David Evans Research Centre, Nottingham University Hospitals, Nottingham, UK; 8 Department of Radiology, Royal Brompton Hospital, London, UK; 9 National Heart and Lung Institute, Imperial College London, London, UK

**Keywords:** Lung neoplasms, Early detection of cancer, Mass screening, Radiology, Solitary pulmonary nodule

## Abstract

**Objectives:**

Successful lung cancer screening delivery requires sensitive, timely reporting of low-dose computed tomography (LDCT) scans, placing a demand on radiology resources. Trained non-radiologist readers and computer-assisted detection (CADe) software may offer strategies to optimise the use of radiology resources without loss of sensitivity. This report examines the accuracy of trained reporting radiographers using CADe support to report LDCT scans performed as part of the Lung Screen Uptake Trial (LSUT).

**Methods:**

In this observational cohort study, two radiographers independently read all LDCT performed within LSUT and reported on the presence of clinically significant nodules and common incidental findings (IFs), including recommendations for management. Reports were compared against a ‘reference standard’ (RS) derived from nodules identified by study radiologists without CADe, plus consensus radiologist review of any additional nodules identified by the radiographers.

**Results:**

A total of 716 scans were included, 158 of which had one or more clinically significant pulmonary nodules as per our RS. Radiographer sensitivity against the RS was 68–73.7%, with specificity of 92.1–92.7%. Sensitivity for detection of proven cancers diagnosed from the baseline scan was 83.3–100%. The spectrum of IFs exceeded what could reasonably be covered in radiographer training.

**Conclusion:**

Our findings highlight the complexity of LDCT reporting requirements, including the limitations of CADe and the breadth of IFs. We are unable to recommend CADe-supported radiographers as a sole reader of LDCT scans, but propose potential avenues for further research including initial triage of abnormal LDCT or reporting of follow-up surveillance scans.

**Key Points:**

• *Successful roll-out of mass screening programmes for lung cancer depends on timely, accurate CT scan reporting, placing a demand on existing radiology resources.*

• *This observational cohort study examines the accuracy of trained radiographers using computer-assisted detection (CADe) software to report lung cancer screening CT scans, as a potential means of supporting reporting workflows in LCS programmes.*

• *CADe-supported radiographers were less sensitive than radiologists at identifying clinically significant pulmonary nodules, but had a low false-positive rate and good sensitivity for detection of confirmed cancers.*

**Supplementary Information:**

The online version contains supplementary material available at 10.1007/s00330-022-08824-1.

## Introduction

Lung cancer is the commonest cause of cancer-associated death worldwide (1), largely due to patients presenting with disease which is already at an advanced stage. Large randomised controlled trials have demonstrated that lung cancer screening (LCS) using low-dose computed tomography (LDCT) enables diagnosis at an early stage and saves lives, reducing lung cancer–specific mortality by between 20 and 29% (2–4). There is now motivation to introduce LCS programmes worldwide, and within the UK several targeted LCS programmes are underway funded by NHS England (5). However, wide-scale delivery of LCS requires measures to ensure efficient use of resources and limit costs. In particular, timely and accurate LDCT interpretation places a significant demand on radiology resources, and novel approaches towards LDCT reporting workflows are needed.

Computer-aided detection (CADe) software is increasingly used by thoracic radiologists to support LDCT reporting through automated nodule detection, and is considered a minimum software requirement in the targeted LCS programme in England (5). This considerably reduces the time taken for scan interpretation (6) and improves sensitivity compared to both single- and double-radiologist reporting (7, 8). However, the specificity of CADe is limited, requiring interpretation by an experienced reader to discount false positives (9) and to report other clinically significant incidental findings (IFs) (10).

Trained radiographers have an existing role in chest radiograph (CXR) interpretation (11–13). Previous studies report additional first reading of a CT by a radiographer can improve both read times and sensitivity for radiologists (14) and that the highest performing radiographers may have comparable or even better accuracy than some thoracic radiologists (15). Furthermore, specifically trained technicians using CADe support have shown good accuracy in identifying pulmonary nodules measuring > 1 mm (16). Appropriately trained radiographers may have a role in supporting LCS reporting workloads; however, their ability to interpret the clinical significance and recommend management of LDCT findings has not previously been reported.

This study evaluates the ability of radiographers with CADe support to distinguish normal from abnormal CTs, and the accuracy of radiographers’ LDCT interpretation, including the detection and evaluation of nodules and lung masses, common IFs, and recommendations for management.

## Methods

### Study population and case selection

This study is an a priori sub-study of the Lung Screen Uptake Trial (LSUT) (17). LSUT recruited individuals aged 60–75 years to undergo a single round of LDCT screening if meeting either the United States Preventive Services Task Force (USPSTF) threshold (≥ 30 pack years smoking history, and quit ≤ 15 years ago), or the Liverpool Lung Project (LLP) or the Prostate Lung Colorectal and Ovarian (PLCO_m2012_) lung cancer risk model thresholds (≥ 2.5% over 5 years and ≥ 1.51% over 6 years respectively) (18). All LDCT scans performed in LSUT were made available for use in this sub-study.

### Scan techniques

Scans were performed via a sixteen channel or higher multi-detector, non-ECG-voltage-gated CT without intravenous contrast. The lung parenchyma was scanned from apices to bases in a single craniocaudal acquisition during suspected maximal inspiration, with a field of view selected as the smallest diameter required to accommodate the entire lung parenchyma. Thin detector collimation (0.5 mm) was used. Images were reconstructed at 0.5–1.0 mm section thickness using standard soft tissue and lung algorithms. Radiation exposures were as low as possible whilst maintaining good image quality. The tube potential and current-time product varied between 80–120 kVp and 20–80 mAs respectively according to participant body habitus.

### LDCT reporting and management

All scans were reported by radiologists (certified by the UK Royal College of Radiology) with 5–28 years of expertise in thoracic imaging. Radiologists viewed scans using local Picture Archiving and Communication System (PACS) programmes. Any identified nodules were measured manually by maximum diameter, with automated volumetry (Vitrea Advanced Visualization Platform, Canon Medical Systems Ltd, version 6.7.4, or Carestream Health, Philips Medical, version 12.1) available via a separate workstation.

Radiologists completed a structured electronic report for each scan, capturing the total number of nodules, detailed descriptions of up to two nodules (diameter, volume, density, location, and morphology), the presence or absence of other pulmonary and extra-pulmonary IFs, and recorded scan reading times. Emphysema, coronary artery calcification (CAC), interstitial lung abnormalities (ILAs), and bronchiectasis were quantified visually as none, mild, moderate, or severe. Expected management of pulmonary nodules was broadly in accordance with the British Thoracic Society (BTS) guidelines (19), but allowances were made for radiologists’ clinical judgment. Five percent of scans were re-reported by a radiologist at the alternate site for quality assurance (QA) purposes, with discrepant reports reviewed a third radiologist (A.N.) for consensus.

### Radiographer training and reporting

Two radiographers (denoted as R1 and R2) experienced in thoracic CT acquisition and with prior qualification in chest radiograph reporting, but without prior experience in thoracic CT reporting, underwent supervised interpretation on a series of 50 ‘training’ CT scans containing pulmonary nodules followed by assessed interpretation of a further seven scans. Additional training covered principles of lung cancer screening, common IFs (such as emphysema, CAC, ILAs, and bronchiectasis) and pulmonary nodule management guidelines. On completion of training, all LSUT scans were made available via a CADe workstation (Veolity^TM^ MeVis Medical Solutions AG, version 1.2). Both radiographers completed identical LDCT reporting electronic reports to those used by the radiologists and either recommended management plans or deferred to a radiologist for advice.

### Study outcomes

#### Determination of nodule concordance

Radiographer and radiologist reports with matching descriptions of ≥ 1 pulmonary nodule warranting referral to a lung cancer tumour board or further CT surveillance were considered in agreement. Lobar location, diameter (to within 2 mm, allowing for differences in inter-observer measurement (20)), and morphology — solid (SN), part-solid (PSN), or ground glass (GGN) — were all required for a match to be considered present. Where matches could not be assessed from the reported measurements, the original images were viewed in Veolity^TM^ and PACS by the authors (H.H., M.R., A.N.) to determine the likelihood of agreement. Where radiographers deferred to a radiologist, the radiographer report was interpreted as being in agreement with the original reporting radiologist for the purposes of analysis.

#### Derivation of ‘reference standard’ (RS)

**Fig. 1:**
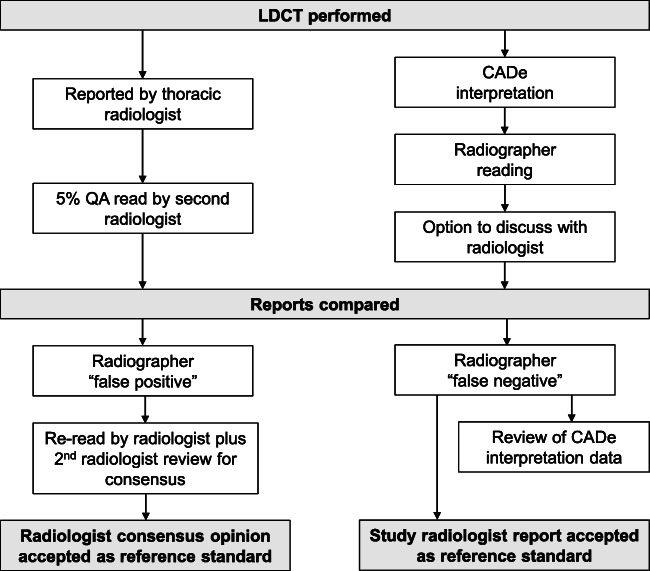
Derivation of reference standard

The ‘reference standard’ (RS) comprised of either the original radiologist report or the final consensus opinion of any re-reviewed scans (Fig. 1). To determine the RS, radiographer reports were first compared against the original reporting radiologist (P.S., M.T., A.A., S.B., M.J.S.) and, where performed, QA second-read reports. Where a radiographer reported a pulmonary nodule not documented by the radiologist, the scan was re-reviewed by a second radiologist (A.N.) for consensus. Outstanding discrepancies were reviewed by both the original reading radiologist and a third radiologist (A.D.) for a final opinion. Neither of the radiologists A.D. or A.N. participated as readers in the original study. Where radiologist reports were influenced by previous imaging inaccessible to radiographers, BTS guidelines were used to dictate expected action from radiographers. Nodules managed outside BTS recommendations at the radiologists’ discretion (e.g. sub-5-mm diameter with concerning morphology) were excluded from the RS.

Compared to the RS, any scans reported by a radiographer as a ‘false negative’ were re-reviewed by the authors (H.H., M.R.) on a Veolity^TM^ workstation, and categorised by whether they were detected and discounted by the radiographer or missed altogether, with reasons for discounting described where applicable.

Primary outcomes were defined on a per-scan basis as follows:

- *Relative sensitivity and specificity* — for correct identification of scans as containing one or more nodule(s) or suspicious lesion(s) warranting CT surveillance or lung tumour board review, relative to the RS

Additional secondary outcomes of interest were as follows:

- *Sensitivity for identification of confirmed cancers —* including cancers detected either from the baseline scan or following nodule surveillance
- *Proportion of scans deferred for a radiologist opinion*
- *Identification of common IFs* including coronary artery calcification (CAC) and emphysema, compared against the original radiologist report
- *Concordance with BTS nodule management guidelines* — including the proportion of scan reports which recommended either more or less intensive follow-up compared to BTS recommendations and the number of cancers detected from these scans (see Supplementary material table e4)
- *Self-reported read times* comparing each radiographer against pooled radiologist reading times (see Supplementary materials figure e1)

### Statistical analysis

The present analysis was pre-specified in the LSUT statistical analysis plan (available at: https://osf.io/9kuzb/, Appendix 7.1). Minor deviations from this are described in Supplementary materials 6. Demographic and clinical characteristics are reported with descriptive statistics. Relative sensitivity/specificity was calculated for each radiographer for correct identification of a ‘positive’ or ‘negative’ scan as per the RS, as well as for confirmed cancers. Relative sensitivities, rates of deferral to a radiologist, and concordance versus divergence from management guidelines between radiographer readers (Supplementary materials table e4) were compared using McNemar’s test. Self-reported read times were compared using Wilcoxon rank sum test. Inter-observer agreement in assessment of IFs was compared using weighted Cohen’s kappa (*K*_w_), with levels of agreement interpreted as per Landis and Koch guidelines (21). Statistical analyses were performed using STATA (v 15.1). Analyses were two-sided and *p* values < 0.05 were considered statistically significant.

## Results

### Study population

A total of 770 participants enrolled in LSUT and underwent a single round of LDCT screening, with 771 scans performed (one participant was recalled for a repeat CT due to technical inadequacy). Participant demographics are summarised in Supplementary Materials table e1. Thirty-six lung cancers were diagnosed after a median 1044 days follow-up (17).

### Derivation of reference standard

Including QA reads, study radiologists identified 30 ‘suspicious lesions’ requiring MDT referral. A total of 133 scans (17.3%) were identified as containing ≥ 1 ‘indeterminate nodule’, of which eight were discounted following comparison with historical imaging. Nineteen scans were excluded from the RS as all nodules fell below the BTS guideline size-threshold for warranting surveillance. Thirty-five scans had non-nodular opacities (e.g. focal fibrosis, cystic lesions, consolidation) warranting radiological surveillance, one of which resulted in a cancer diagnosis. The remaining 572 scans had no clinically significant nodules identified.

R1 and R2 completed reports for 95.2% (733/770) and 98.7% (760/770) of scans respectively. Reasons for unreported scans included concerns regarding technical inadequacy (R1 = 8, R2 = 3), issues importing images for viewing (R1 = 20, R2 = 7), or issues with CADe software (R1 = 9). R2 chose to read scans without CADe nodule interpretation where CADe processing failure was the only technical issue (18 scans) but this was not mandated.

**Fig. 2:**
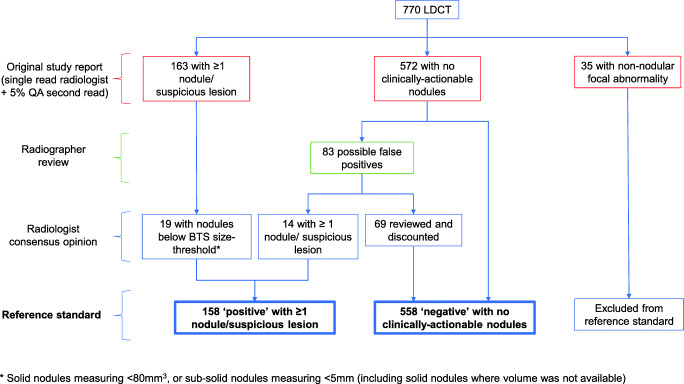
Reporting process and reference standard

Initial review of radiographer reads against single-read/QA radiologist reports identified 83 possible ‘false-positives’. On radiologist consensus review, 13.2% (11/83) were considered to be a significant nodule and 83.1% (69/83) were considered non-significant due to benign morphological appearances (53/69) or small size (16/69). In the other three cases, the findings reported by the radiographer were deemed non-significant but the reviewing radiologist identified other nodules that had been missed or dismissed by both the radiographers and the original reporting radiologist. All newly identified nodules underwent further imaging review, with none demonstrating interval growth. The final RS consisted of 716 scans: 158 ‘positive’ scans with ≥ 1 nodule or suspicious lesion, and 558 ‘negative’ scans with no actionable nodules (Fig. 2).

### Primary outcome: relative sensitivity and specificity

Relative sensitivity for identification of ‘positive’ scans was 68.0% for R1 and 73.7% for R2, with relative specificity of 92.1% and 92.7% and false-positive rates of 7.9% and 7.3% respectively (Table 1). There was no statistically significant difference in relative sensitivity between R1 and R2 (*p* = 0.732), but both radiographers had significantly lower sensitivity than the study radiologists (*p* < 0.001 for both). Additional post hoc analysis demonstrated improved sensitivity (76.2% vs 68.0% and 79.2% vs 73.7% for R1 and R2 respectively) if the nodule positivity threshold was adjusted from 5 to 6 mm (Supplementary materials table e2).

**Table 1:** Relative sensitivity/specificity and false-positive rates for nodule detection, against reference standard

	R1	R2	Study radiologists^a^
Relative sensitivity	68.0% (102/150^b^)	73.7% (115/156^b^)	91.1% (144/158)
Relative specificity	92.1% (490/532^b^)	92.7% (510/550^b^)	NA
False-positive rate	7.9% (42/532^b^)	7.3% (40/550)	NA

^a^Includes single-reader and QA outcomes, prior to re-review and consensus process

^b^Denominator excludes scans where reports could not be issued by the radiographer

Sixty-one ‘positive’ scans in the RS were reported as ‘false negative’ by one or both radiographers (R1 = 43, R2 = 38). Of these 61 scans, 37 scan containing 40 nodules (7 of which proved malignant) were reviewed in Veolity^TM^ by the authors (HH and MR) to assess factors contributing towards false-negative outcomes (Table 2). The remaining 24 scans were no longer accessible via the Veolity^TM^ workstation. Twenty nodules were not identified by CADe (seven SN, three PSN, and 10 GGN), four of which proved malignant. 15.1% (5/33, R1) and 16.0% (4/25, R2) of ‘false negatives’ were discounted by the radiographers on account of size below the BTS guideline threshold.

**Table 2:** Breakdown of the 40 nodules available for review that were categorised as false negative by one or both radiographers

			R1	R2
True positive			**5**	**15** 5 cancers
False negative			**33**	**25**
Identified by CADe, discounted by radiographer	*Discounted on size*	*Diameter < 5 mm*	*1*	*1*
		*Volume < 80 mm*^*3*^ *(SN*^a^ *only)*	*4*	*3*
	*Discounted on morphology*		*7* *1 cancer*	*6*
	*Unable to establish reason for discounting*		*3* *1 cancer*	*5* *1 cancer*
Not identified by CADe	*Not identified by radiographer*		*15* *2 cancers*	*10* *1 cancer*
	*Identified by radiographer, discounted on morphology*		*3* *2 cancers*	*0*
Scan not reported			**2** 1 cancer	**0**
Total nodules			**40**	**40**

^a^*SN* solid nodule

### Secondary outcomes

#### Sensitivity for confirmed cancers

Within scans included in the RS, 18 cancers were detected following direct MDT referral from the baseline scan, for which sensitivity was 83.3% (15/18) for R1 and 100% (17/17) for R2. Of the 15 cancers diagnosed during nodule surveillance, 69.2% (9/13) for R1 and 86.7% (13/15) for R2 were identified from the baseline scan.

#### Proportion of scans deferred for radiologist review

There was a significant difference between radiographers as regards the proportion of scans deferred for radiologist discussion, at 6.5% (48/733) and 10.8% (82/760) for R1 and R2 respectively (*p* = 0.015).

#### Identification of common IFs

Inter-observer agreement between radiographers and radiologists for graded severity of emphysema was ‘good’ for R1 (*K*_w_ 0.61) and ‘fair’ for R2 (*K*_w_ 0.54) respectively (*p* < 0.001 for both). For CAC, the level of agreement was ‘fair’ for R1 (*K*_w_ 0.44) and ‘good’ for R2 (*K*_w_ 0.74) respectively (*p* < 0.001 for both). Other benign pulmonary pathologies including usual interstitial pneumonitis (UIP) and non-UIP type interstitial lung disease and bronchiectasis were reported with low frequency (see Supplementary materials Table e3), so were omitted from further analysis. The variety of other extra-pulmonary pathologies reported by radiologists was considered too broad to reasonably expect radiographers to detect and interpret, as their training did not cover such pathologies.

## Discussion

We report the accuracy of CADe-assisted radiographer reading of prevalence round LCS LDCT scans, compared to a RS defined by radiologist reports +/− consensus review. CADe-assisted radiographers achieved a high specificity for identifying positive scans, but their relative sensitivity for the detection of positive findings was 68–74% (false-negative rate of 26–32%). The level of agreement for the two most common intrathoracic IFs — emphysema and CAC — was fair to good, but the frequency of other IFs (bronchiectasis and ILAs) was too low to permit analysis of radiographer performance, and the broad range of other IFs exceeded what could reasonably be addressed in radiographer training. These last two findings potentially counteract the benefit of the low false-positive rate and suggest that, at least for the foreseeable future, a radiographer in conjunction with CADe cannot be the sole reader of an LDCT.

Ritchie et al report excellent accuracy (sensitivity and specificity both > 90%) in a specifically trained technician’s ability to identify non-calcified nodules measuring > 1 mm in diameter using CADe support (16) but did not evaluate their ability to distinguish the clinical significance of such nodules, avoiding some of the uncertainty faced by our radiographers and lessening the impact of inter-observer measurement variation on radiographer accuracy. The radiographer sensitivities reported by Nair et al are closer to those observed in this study at 61.4–79.4%, though this was without the use of CADe (14). However, whilst our findings preclude CADe-assisted radiographers as a single reader of LDCT, the relatively high detection rate for suspicious cancers (83.3–100%) and low false-positive rates reported here suggest that CADe-assisted, radiographer-led triage of LDCT for radiologist review would be a feasible strategy. Future research could examine whether such a strategy would save time compared to CADe-assisted radiologist reading, but in this study both radiographers achieved significantly faster read times than radiologists reading without CADe (Supplementary materials figure e1). Alternatively, radiographers may have a role in reporting follow-up studies, which in clinically indicated nodule surveillance scans has improved clinical service delivery with significant cost- and time-saving benefits (22). However, this would require further research to evaluate radiographer accuracy in the comparison of current and prior imaging and interpretation of longitudinal nodule measurements, and particularly in the detection of new nodules, given the increased risk of lung cancer in such findings (23). Radiographers had acceptable agreement for evaluation of emphysema and CAC; however, reporting of these findings is a contentious issue in LCS. Although higher CAC on LDCT is associated with cardiovascular or all-cause mortality (24), the impact of acting on these findings on patient outcomes has not been conclusively demonstrated (25). Refining the management of such findings (26, 27) with appropriate training could improve radiographer performance with this regard as well as improve the efficiency of screening and downstream impact on other health care resources.

This sub-study includes virtually all LDCTs performed as part of LSUT and is therefore highly representative of UK-based LCS populations, allowing evaluation of radiographer accuracy in both identifying significant nodules/masses and reducing false-positives, both key for successful LCS programmes. However, the current study has limitations. Firstly, only two of the intended three radiographers were able to participate and given the observed variation in performance, additional readers may have been valuable in reducing this spread. There was no capacity for radiologist feedback to radiographers, which other studies suggest may have improved radiographer performance (16). We were unable to examine sensitivity on a per-nodule basis, and findings such as nodular consolidation and other focal abnormalities were not formally covered in radiographer training, preventing us from including such scans in our RS. We also acknowledge that consensus radiologist reads of all scans may have identified additional false positive/false negatives within our reference standard, though this would only have further increased the radiographer false-negative rate. Finally, the techniques with which nodules were measured by radiologists (manual diameter, then automated volume as indicated) and radiographers (automated diameter/volume for all) may have contributed to differences in interpretation of clinical significance. As nodules felt to be non-significant were not consistently described in detail by all readers, we are limited in our ability to detect such instances from the available data. However, a significant minority of false negatives were due to differences in radiographer versus radiologist measurement above/below BTS thresholds, and a post hoc analysis examining detection of nodules ≥ 6 mm in diameter showed improved sensitivity for both radiographers (Supplementary table e2).

Radiographers in our study achieved a relatively low level of false-positives, but the relative sensitivity for positive findings (including confirmed cancers) was too low to allow recommendation of CADe-assisted radiographer reading as a strategy more widely. Future research is recommended to examine alternative roles for radiographers in supporting LCS delivery at a population level.

## Supplementary Information

ESM 1 (DOCX 79 kb)

## Abbreviations

BTS: British Thoracic Society
CAC: Coronary artery calcification
CADe: Computer-assisted detection
GGN: Ground glass nodule
IF: Incidental finding
ILA: Interstitial lung abnormalities
LCS: Lung cancer screening
LDCT: Low-dose computed tomography
LLP: Liverpool Lung Project
LSUT: Lung Screen Uptake Trial
MDT: Multidisciplinary team
PACS: Picture Archiving and Communication System
PLCO: Prostate Lung Colorectal Ovarian
PSN: Part-solid nodule
QA: Quality assurance
RS: Reference standard
SN: Solid nodule
USPSTF: United States Preventive Services Task Force

## Declarations

### Guarantor

The scientific guarantor of this publication is Professor Sam Janes.

### Statistics and biometry

Professor Stephen Duffy kindly provided statistical advice for this manuscript.

### Study subjects or cohorts overlap

Some study subjects or cohorts have been previously reported in previous publications: Data relating to the response to targeted LCS information materials (Quaife, AJRCCM, 2020) and lung cancer prevalence (Ruparel, Thorax, 2020) in this cohort have been published previously. Additionally, preliminary analyses from this paper were presented as a poster at the British Thoracic Conference 2019, with the abstract published (Hall, Thorax 2019). The full analysis of radiographer reporting presented in this manuscript has not been published elsewhere.

### Methodology

• prospective

• observational

• multicenter study
