# Supplementary material for: The role of computer-assisted radiographer reporting in lung cancer screening programmes
Source: Eur Radiol. 2022 May 14;32(10):6891–9. doi: 10.1007/s00330-022-08824-1 (PMC9474336; doi:10.1007/s00330-022-08824-1)
Supplement: Supplementary file 1 — (DOCX 79 kb) [file 330_2022_8824_MOESM1_ESM.docx]

**The role of computer-assisted radiographer reporting in lung cancer screening programmes – supplementary materials**

H Hall, M Ruparel, S Quaife, JL Dickson, C Horst, S Tisi, J Batty, N Woznitza, A Ahmed, S Burke, P Shaw, MJ Soo, M Taylor, N Navani, A Bhowmik, DR Baldwin, SW Duffy, A Nair, A Devaraj, SM Janes.

Supplementary materials.

1. Table e1 - Participant demographics
2. Table e2 – Relative sensitivity for detection of positive scans containing nodules ≥6mm in maximum diameter
3. Table e3 - Incidental findings
4. Table e4 - Concordance of management decisions against BTS guidelines
5. Figure e1: Self-reported read times for radiologists versus radiographers
6. Deviations from statistical analysis plan

Table e1: Participant demographics

|  | Positive  (n = 158) | Negative  (n = 558) | other  (n = 35) |
| --- | --- | --- | --- |
| Male  Age* | 93 (58.9%)  67 (63, 69) | 305 (54.7%)  66 (63, 69) | 18 (51.4%)  65 (62, 68) |
| Ethnicity  White  black  other | 132 (83.5%)  13 (8.2%)  13 (8.2%) | 464 (83.2%)  58 (10.4%)  36 (6.4%) | 32 (91.4%)  3 (8.6%)  0 |
| IMD quintile  1  2  3  4  5  Missing | 89 (56.3%)  53 (33.5%)  2 (1.3%)  1 (0.6%)  0  13 (8.2%) | 309 (55.4%)  185 (33.2%)  16 (2.9%)  1 (0.2%)  0  47 (8.4%) | 12 (34.3%)  14 (40%)  0  0  0  9 (25.7%) |
| Smoking status  Current  Former  Pack years * | 119 (75.3%)  39 (24.7%)  41 (27,56) | 402 (72.0%)  156 (28.0%)  38 (25,50) | 23 (65.7%)  12 (34.3%)  42 (34, 80) |
| FEV1 Absolute*  FEV1 % predicted*  FEV1:FVC* | 1.99 (1.51, 2.49)  77.5 (61.5, 91.5)  67 (58.5,74) | 2.06 (1.65,2.6)  82 (68, 96)  69 (62,75) | 1.77 (1.28, 2.44)  80.5 (53, 90)  61.5 (54, 67) |
| WHO PS  0  1  2  3-4 | 136 (86.1%)  21 (13.3%)  1 (0.6)  0 | 510 (91.4%)  41 (7.4%)  7 (1.3%)  0 | 25 (71.4%)  10 (28.6%)  0  0 |
| LLP *  PLCO_m2012_ *  Follow up (days)* | 5.6 (3.8,9.7)  4.3 (1.8, 8.9)  834 (675, 956) | 5.4 (3.8, 8.5)  3.6 (1.7, 6.8)  863 (708, 1001) | 7.7 (4.4, 8.9)  6.0 (3.4, 11.2)  911 (778, 1046) |

* Includes those with non-nodular findings requiring CT surveillance

** Expressed as median (IQR)

Table e2. Relative sensitivity for detection of positive scans containing nodules ≥6mm in maximum diameter (n=132) compared to nodules ≥5mm (n=158)

|  | R1 | R2 | Study radiologists^a^ |
| --- | --- | --- | --- |
| Relative sensitivity at ≥5mm threshold | 68.0% (102/150^b^) | 73.7% (115/156^b^) | 91.1% (144/158) |
| Relative sensitivity at ≥6mm threshold | 76.2% (76/126 ^b^) | 79.2% (103/130 ^b^) | 93.2% (123/132) |

^a^ Includes single-reader and QA outcomes, prior to re-review and consensus process

^b^ Denominator excludes scans where reports could not be issued by the radiographer

Table e3. Rates of incidental findings

|  | Total reported frequency* | Mild | Moderate | Severe |
| --- | --- | --- | --- | --- |
| CAC | 64.5% | 245 | 175 | 77 |
| Emphysema | 61.2% | 286 | 143 | 42 |
| ILA (UIP-type) | 2.3% | 14 | 4 | 0 |
| ILA (non-UIP) | 4.7% | 31 | 5 | 0 |
| Bronchiectasis | 47.1% | 324 | 37 | 2 |

CAC – coronary artery calcification; ILA = Interstitial lung abnormalities; UIP = usual interstitial pneumonia

* From original study radiologist report

Table e4: Concordance of management decisions against BTS guidelines

|  | | R1 | R2 | Radiologists |
| --- | --- | --- | --- | --- |
| Total nodules (scans) | | 229 (175) | 221 (174) | 245 (196) |
| Scans included* | | 131** | 150** | 155*** |
| % Concordant | | 52 (39.7%)  *15 cancers* | 91 (60.7%)  *22 cancers* | 111 (71.6%)  *28 cancers* |
| % Divergent | More active follow-up | 26 (19.8%)  *1 cancer* | 35 (23.3%)  *4 cancers* | 22 (14.2%)  *3 cancers* |
|  | Less active follow-up | 53 (40.5%)  *3 cancers* | 24 (16%)  *1 cancer* | 20 (12.9%)  *1 cancer* |

* Includes all scans with at least one measured nodule of defined solid, part-solid or pure ground glass morphology, and recommendation for either MDT referral, defined CT surveillance interval or no radiological follow up based on the dominant nodule

** Excludes scans deferred to radiologist for opinion

*** Excludes scans where management was led by review of previous imaging or superseded by an incidental finding

Figure e1: Comparison of self-reported read times between radiographers and radiologists

Read-times were available for 753 (97.8%) of radiologist reports, 738 (95.8%) of reports by R1 and 754 (97.9%) by R2. Radiologists recorded significantly longer and more variable read times than either radiographer, with median read-time 10 minutes (IQR 5,15) versus 3 minutes, IQR (2,5) for radiologist versus R1 and 5 minutes, IQR (4,8) for radiologist versus R2, (p<0.001 for both comparisons).


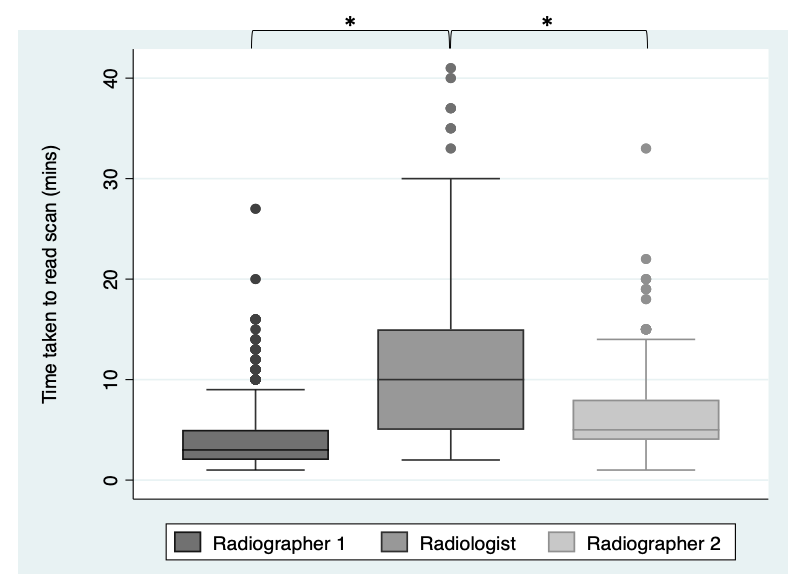


Deviations from statistical analysis plan

- Our initial proposal was to include three reporting radiographers in the study. Three radiographers completed training however one withdrew from the study at that point due to personal circumstances.
- The arbitrating radiologist for ‘false positives’ was a 3^rd^ independent radiologist rather than original radiologist reader as planned. This is largely due to staffing availability at the time of carrying out the study, though also was felt to be a superior strategy than utilising the original reader for this task.
- Due to the need to deliver timely clinical reports as part of study delivery, it was not feasible to report each scan on a per nodule basis. Particularly given that clinical management recommendations are made on a per scan basis based on the dominant nodule, a decision was therefore made to revise the approach to a per-scan basis focusing on up to two most dominant nodules.
- The secondary outcomes reported on in this paper are different to the proposed secondary outcomes in the SAP as follows
  - Brock score was not included in the analysis due to difficulties with the radiologist data in having complete brock scores for the whole dataset, and also as radiologists did not have access to the same software as the radiographers when reporting the nodules and so often manually measured many of the nodules resulting in variable brock scores that we felt were not suitable for comparison in this study
  - A analysis of the cost of reading was planned to be included as this would be lowered by the time and reduced cost of utilising radiographers rather than radiologists, however, as our recommendation has been to not employ this strategy, we have not explored the relative costs in this paper
